# Advanced pharmacodynamics of cangrelor in healthy volunteers: a dose-finding, open-label, pilot trial

**DOI:** 10.1186/s12959-022-00377-z

**Published:** 2022-04-14

**Authors:** Georg Gelbenegger, Juergen Grafeneder, Gloria M. Gager, Jolanta M. Siller-Matula, Michael Schwameis, Bernd Jilma, Christian Schoergenhofer

**Affiliations:** 1 Department of Clinical Pharmacology, Medical University of Vienna, Waehringer Guertel 18-20, 1090 Vienna, Austria; 2 Department of Emergency Medicine, Medical University of Vienna, Vienna, Austria; 3 Department of Medicine II, Division of Cardiology, Medical University of Vienna, Vienna, Austria; 4 Department of Experimental and Clinical Pharmacology, Centre for Preclinical Research and Technology (CEPT), Medical University of Warsaw, Warsaw, Poland

**Keywords:** Platelet inhibition, Cangrelor, Prehospital, P2Y12, Pharmacodynamics, Myocardial infarction, STEMI

## Abstract

**Background:**

High on-treatment platelet reactivity (HTPR) remains a major problem in the acute management of ST-elevation myocardial infarction (STEMI), leading to higher rates of stent thrombosis and mortality. We aimed to investigate a novel, prehospital treatment strategy using cangrelor and tested its pharmacodynamic effects in a model using healthy volunteers.

**Methods:**

We conducted a dose-finding, open-label, pilot trial including 12 healthy volunteers and tested three ascending bolus infusions of cangrelor (5 mg, 10 mg and 20 mg) and a bolus infusion followed by a continuous infusion via an intravenous (IV) flow regulator. Platelet function was assessed using multiple electrode aggregometry (MEA), vasodilator-stimulated phosphoprotein phosphorylation assay (VASP-P) and the platelet function analyzer. In an ex vivo experiment, epinephrine was used to counteract the antiplatelet effect of cangrelor.

**Results:**

All cangrelor bolus infusions resulted in immediate and pronounced platelet inhibition. Bolus infusions of cangrelor 20 mg resulted in sufficient platelet inhibition assessed by MEA for 20 min in 90% of subjects. Infusion of cangrelor via the IV flow regulator resulted in sufficient platelet inhibition throughout the course of administration. Ex vivo epinephrine, in concentrations of 200 and 500 ng/mL was able to partially reverse the antiplatelet effect of cangrelor in a dose-dependent manner.

**Conclusions:**

Weight-adapted bolus infusions followed by a continuous infusion of cangrelor via IV flow regulator result in immediate and pronounced platelet inhibition in healthy subjects. Cangrelor given as weight-adapted bolus infusion followed by a continuous infusion using an IV flow regulator may be a viable treatment approach for effective and well controllable prehospital platelet inhibition.

**Trial registration:**

EC (Medical University of Vienna) 1835/2019 and EudraCT 2019-002792-34.

**Supplementary Information:**

The online version contains supplementary material available at 10.1186/s12959-022-00377-z.

## Introduction

ST-elevation myocardial infarction (STEMI) makes up about 30% of acute coronary syndromes, with substantial in-hospital mortality of 4–12% [1, 2]. Acute STEMI care involves dual antiplatelet therapy, including intravenous aspirin and an orally administered P2Y_12_ inhibitor (clopidogrel, prasugrel or ticagrelor) [3, 4]. Early administration of antiplatelet therapy at time of diagnosis, in particular, is recommended as it is associated with a decreased incidence of stent thrombosis in patients presenting with STEMI [5]. Insufficient pharmacodynamic response to antiplatelet therapy is termed high on-treatment platelet reactivity (HTPR), which is associated with an increased risk of major adverse cardiovascular events and stent thrombosis after percutaneous coronary intervention (PCI) [6, 7]. Patients presenting with STEMI should undergo emergency PCI within 120 min (min) calculated from time of STEMI diagnosis to wire crossing, at which time point, ideally, platelet function is already fully inhibited.

Substantially high rates of HTPR in STEMI patients have been detected across all three P2Y_12_ inhibitors 2 h after loading dose (up to 64.5% in clopidogrel, 35% in prasugrel and 50% in ticagrelor) [8–10]. In addition, the use of morphine in STEMI patients further contributes to a decreased pharmacodynamic response to antiplatelet therapy due to a delayed absorption of oral P2Y_12_ inhibitors [11, 12]. The striking frequency of HTPR after antiplatelet treatment in the acute setting of STEMI prompts the search for new treatment strategies aiming to overcome HTPR and its consequences.

Cangrelor is a rapid-onset, short-acting and reversible non-thienopyridine antiplatelet agent targeting the ADP-activated P2Y_12_ receptor. It is the first intravenous P2Y_12_ inhibitor with a relatively short half-life of 3–6 min, therefore usually given as a continuous infusion to maintain antiplatelet activity.

The prehospital use of cangrelor as a fixed-dose bolus infusion or administered via an IV flow regulator may be able to achieve a rapid onset of sufficient platelet inhibition in acute STEMI patients, resulting in a lower rate of HTPR. To test the feasibility of this novel concept of prehospital cangrelor use, we conducted a prospective, open-label, dose-finding pilot trial of cangrelor in healthy volunteers.

## Methods

The trial was conducted at the Department of Clinical Pharmacology at the Medical University of Vienna, between December 2019 and January 2020, and was performed in accordance with the Good Clinical Practice guideline and the principles set forth in the Declaration of Helsinki. The independent Ethics Committee of the Medical University of Vienna and the national competent authority (Austrian Agency for Health and Food Safety) approved the trial. The trial was registered at the EudraCT database under the identifier 2019-002792-34. Written and oral informed consent was obtained from all healthy volunteers prior to any trial-related activity. The trial protocol is available upon request to the corresponding author.

### Participants

Twelve healthy volunteers aged between 18 and 74 years without any relevant medical history, and with normal findings in their physical examination and baseline laboratory results were included in this trial. Major exclusion criteria included active bleeding or increased risk of bleeding because of known coagulation or platelet disorders, current intake of drugs interfering with coagulation or platelets or in the past 30 days and known intolerance or allergy to cangrelor or another P2Y_12_ inhibitor.

### Trial design

This was a prospective, open-label, dose-finding, pilot trial in healthy volunteers. More information about the rationale behind the chosen fixed-dose bolus infusions of cangrelor is provided in the supplementary appendix. Subjects reported to the ward in the morning of the study day after an overnight fast. Two peripheral venous accesses were installed, one for blood sampling and the other for infusion of cangrelor. After the baseline blood sampling, subjects received a bolus dose of cangrelor 5 mg followed by blood samples in short intervals (1 min, 5 min, 10 min, 12.5 min, 15 min, 17.5 min 20 min, 30 min and 60 min) with subsequently performed multiple electrode aggregometry (MEA) analysis. When platelet reactivity returned to baseline (+/− 10%) but not less than 60 min after bolus infusion, the next bolus of cangrelor 10 mg was administered and followed by blood sampling at 1 min, 5 min, 12.5 min, 15 min, 17.5 min, 20 min, 22.5 min, 30 min and 60 min after infusion. Following the same structure, blood samples were taken 1 min, 5 min, 15 min, 17.5 min, 20 min, 22.5 min, 25 min, 30 min and 60 min after bolus infusion of cangrelor 20 mg. The timepoints for blood samples were chosen based on a calculated pharmacokinetic model. When platelet function returned to normal after the third bolus dose of cangrelor, subjects received a fourth bolus dose of 30 μg/kg bodyweight followed by a continuous infusion of 4 μg/kg bodyweight (as recommended by the label) for the duration of 30 min. Blood samples were taken at 1 min, 15 min, 30 min, 45 min and 60 min after bolus infusion.

As an additional experiment, epinephrine, in concentrations of 200 ng/mL and 500 ng/mL, was added to blood samples ex vivo and platelet function was evaluated. This was performed at t_30min_, when a steady state during the continuous cangrelor infusion was reached. The concentrations of 200 ng/mL and 500 ng/mL were chosen based on a calculated pharmacokinetic model of epinephrine concentrations in cardiac arrest.

### Laboratory testing

Routine laboratory testing (including blood chemistry, differential blood count and coagulation) for subject screening and the end-of-study visit was performed by the International Organization for Standardization (ISO) 9002 accredited central laboratory of the University Hospital Vienna.

### Platelet function testing

Platelet function was assessed using multiple electrode aggregometry (MEA), the vasodilator-stimulated phosphoprotein phosphorylation (VASP-P) assay and the platelet function analyzer (PFA-100). The use of different platelet function assays to test for HTPR is recommended [6, 13].

Whole blood aggregometry was determined using the Multiplate analyzer (Dynabyte Medical, Munich, Germany). The system detects the electrical impedance change due to the adhesion and aggregation of platelets on two independent electrode-set surfaces in the test cuvette [14]. A 1:2 dilution of whole blood anti-coagulated with heparin and 0.9% NaCl is stirred at 37 °C for 3 min in the test cuvettes, ADP (adenosine diphosphate, 6.4 μM) is added and the increase in electrical impedance is recorded continuously for 6 min [14]. The mean values of the two independent determinations are expressed in units (U: tenth of area under the platelet aggregation curve, AUC). A good reproducibility of MEA has been reported (< 6% variability) [15]. For ADP-induced platelet aggregation using whole blood MEA, values < 19 U have been classified as low on-treatment platelet reactivity (LTPR), values 19-46 U as moderate on-treatment platelet reactivity (MTPR) and values > 46 U as high on-treatment platelet reactivity (HTPR) [13, 16].

The vasodilator-stimulated phosphoprotein phosphorylation (VASP-P) assay was measured with an enzyme-linked immune assay (CY-Quant VASP/P2Y12 ELISA, REF# 7502, BioCytex, Marceille, France), as described previously [17]. Sodium citrate-anticoagulated whole blood was used [17]. After activation with PGE_1_ or PGE_1_ + ADP, incubation for 10 min and lysis, samples were vortexed and stored at − 20° degrees Celsius. After thawing at room temperature (RT) samples were vortex-mixed. For antigen immobilization, 180 μl of each sample were transferred to the plate and 180 μl dilution buffer were pipetted into blank wells, which were covered and incubated for 30 min at RT. The wells were washed three times with each 300 mL washing solution. For immobilization of immuno conjugate, 200 mL of diluted specified mouse monoclonal antihuman VASP-P ser 239 antibody coupled with peroxidase was added immediately. The wells were covered, incubated again for 30 min at RT and the washing step was repeated. Color development was performed by adding 200 mL tetra-methyl-benzidine and incubating for 5 min at RT. The reaction was stopped with 100 mL H_2_SO_4_ and a 2-min-incubation-step. Within 4 h after stopping the reaction, the absorbance of the reaction product was measured at 450 nm. The platelet reactivity index (PRI) was calculated using optical density (OD) in the presence of PGE_1_ alone or PGE and ADP by means of the formula: PRI (%) = [(OD450_nmPGE1_-OD_450nm(PGE1 + ADP)_/(OD450_nmPGE1_-OD_450nmBlank_)]*100. Calculated values fell sometimes below zero in the ELISA. In this case the values were set to zero for all comparisons. For the VASP-P assay, the cut-off values for LTPR, MTPR and HTPR are defined as a PRI of < 16, 16–50 and > 50%, respectively [6, 18].

Platelet function under high shear rates (5000–6000 s^− 1^) was measured using the platelet function analyzer (PFA-100). Blood samples collected in 3.8% sodium citrate were used. The system measures the time required for the occlusion of the aperture by platelet plugs, termed closure time (CT). The instrument aspirates a blood sample under constant vacuum from the sample reservoir through a capillary and a microscopic aperture (147 μm) cut into the membrane, creating a high shear-induced platelet plug formation [19]. The membrane is coated with a platelet-activating agent, in this study with collagen/adenosine diphosphate (CADP) or collagen/P2Y (CP2Y). The measurement is finished after 5 min. In case of no closure, CT is reported as 301 s. For the PFA (CP2Y test) the recommended cut-off value for HTPR is defined as closure time < 106 s [20].

### Endpoints

The primary endpoint of this study was defined as the time period [minutes] in which sufficient inhibition of ADP-induced platelet activation (LTPR or MTPR) is maintained (≤46 U in MEA analysis). The primary safety endpoint was the incidence of major, minor or minimal bleedings according to the TIMI bleeding definition [21]. Secondary endpoints included pharmacodynamic measurements using MEA, the VASP-P assay and PFA-100.

### Sample size

No formal sample size calculation was performed due to none available data on cangrelor bolus infusion-only. This study can therefore be considered a pilot trial and included 12 healthy subjects. The chosen number of subjects should allow investigating our research questions with enough precision and accuracy to draw conclusions about a possible use in real-world patients [22].

### Statistics

Descriptive statistics was the primary form of analysis of data in this trial. All data is presented using medians and quartiles or means and standard deviations, as applicable. The pharmacodynamic effects were analyzed using standard dose-response curves. The Wilcoxon matched-pairs signed rank test was used for comparison of platelet function between groups in the ex vivo epinephrine experiment. For data analysis, generation of graphs, and calculation of pharmacokinetic parameters, GraphPad Prism (Version 9) and MS Excel were used.

## Results

Twelve healthy subjects were included in this pilot trial. Four subjects were female, eight were male. Subjects had a median age of 32 years and a median weight of 69 kg. The median platelet count was 263 × 10^9^ (IQR 224–281) at study screening and the median ADP-induced platelet aggregation at baseline was 68 U (IQR 59–86) (Table 1).

**Table 1:** Baseline characteristics (on study day) of healthy subjects included in this trial

Baseline characteristics	Median (IQR)	001	002	003	004	005	006	007	008	009	010	011	012
Age - years	32 (24.5–40.5)	24	26	29	41	31	51	39	22	21	56	33	35
Female sex [no.]	4 (8 males)	0	1	1	1	1	0	0	0	0	0	0	0
Weight - kilogram	69 (59–77)	76	58	60	59	58	70	76	68	77	86	64	91,5
Ethnicity	caucasian = c, black = b, asian = a	c	c	c	c	c	b	c	a	c	c	c	c
**Vital parameters**													
RR systolic - mmHg	133 (128–146)	147	107	129	112	136	143	147	128	130	157	140	128
RR diastolic - mmHg	79 (73–88.5)	80	68	78	72	87	97	95	76	78	89	71	82
Heart rate - bpm	79 (70–84)	80	82	61	53	79	87	69	84	78	76	84	74
SpO_2_ - %	98 (97–99)	99	99	100	99	98	95	97	98	98	98	99	97
Temperature (axilla) – degree celsius (°C)	36.0 (35.6–36.1)	35,8	36,4	36	36,1	36,3	35,7	35,5	35	35,5	36,0	36,0	35,9
**PLT count (× 10**^**9**^**)**^**a**^	263 (224–281)	285	291	219	281	280	160	240	247	256	216	270	276
**MEA ADP - U**	68 (59–86)	74	68	96	73	99	39^b^	62	58	60	51	90	68

*PLT* platelet, *MEA* multiple electrode aggregometry, *ADP* adenosine diphosphate, *RR* Riva-Rocci ≈ blood pressure, *bpm* beats per minute, *SpO2* peripheral oxygen saturation, *IQR* interquartile range)

^a^ Measured at screening visit

^b^ Healthy subject 006 presented with an initial ADP-induced platelet aggregation of 39 U, which is already within the therapeutic range. ADP-induced platelet aggregation re-increased to 50 U at 60 min after the first bolus infusion of cangrelor and peaked at 64 U just before the following cangrelor bolus infusion. Potential causes of the initial low level of ADP-induced platelet aggregation could involve a false-low measurement or diurnal variation of platelet function. ADP-induced platelet aggregation is also influenced by variations in platelet count, even within the physiological range [44]

### Platelet function

#### Multiple electrode aggregometry

Intravenous bolus infusion of cangrelor 5 mg effectively decreased ADP-induced platelet aggregation from a median baseline 68 U to a median nadir of 9 U (*p* = 0.0005). The antiplatelet effect (MEA, ADP-induced platelet aggregation < 46 U) was maintained for 17.5 min in all subjects and for 20 min in 9 of 12 subjects (75%). Platelet function returned to normal in all subjects within 60 min.

After bolus infusion of cangrelor 10 mg, ADP-induced platelet aggregation declined rapidly in a similar fashion and adequate platelet inhibition was maintained for 15 min in all subjects, for 20 min in 10 of 12 subjects (83%) and for 22.5 min in 9 of 12 subjects (75%).

In the same way, bolus infusion of cangrelor 20 mg caused instant platelet inhibition and achieved sufficient platelet inhibition for a duration of 15 min in all subjects. Sufficient platelet inhibition was maintained for 20 min in 10 of 11 subjects (90%) and for 25 min in 9 of 11 subjects (82%.)

Cangrelor, when given as a bolus infusion followed by a continuous infusion using an IV flow regulator, resulted in immediate platelet inhibition that was sustained for the complete duration of the continuous infusion in all subjects. Platelet function returned to normal within 30 min after end of infusion in 6 of 11 subjects.

**Fig. 1:**
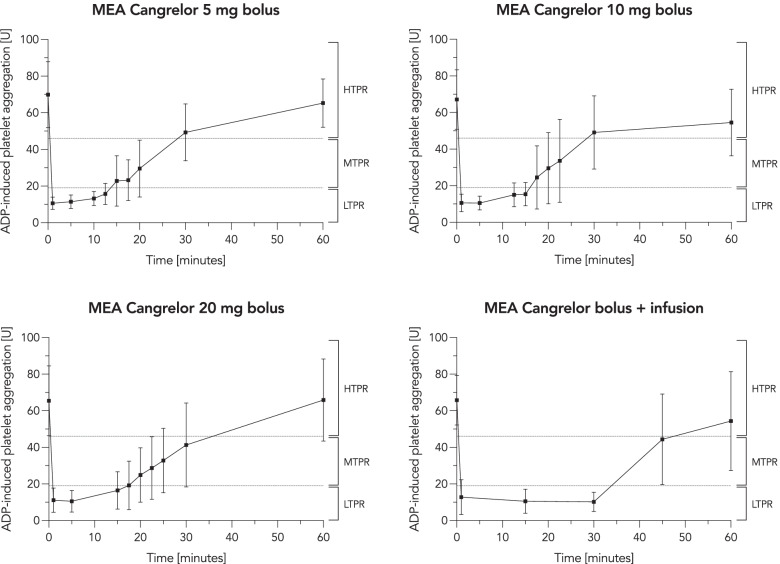
ADP-induced whole blood aggregometry. The four graphs show ADP-induced platelet inhibition over time following cangrelor bolus infusions of 5 mg (upper left panel, *n* = 12), 10 mg (upper right panel, *n* = 12), 20 mg (lower left panel, *n* = 11) and weight-adapted cangrelor bolus infusion followed by a continuous infusion via IV flow regulator (lower right panel, *n* = 11). The dashed lines mark the cut-off values for HTPR, MTPR and LTPR. Moderate platelet inhibition levels of 19–46 U (MTPR) are considered to be within the therapeutic window. Data are shown as mean ± standard deviation. (ADP = adenosine diphosphate, MEA = multiple electrode aggregometry, HTPR = high on-treatment platelet reactivity, MTPR = moderate on-treatment platelet reactivity, LTPR = low on-treatment platelet reactivity)

Graphs showing ADP-induced platelet aggregation over time after cangrelor bolus infusion and infusion via IV flow regulator are shown in Fig. 1.

#### Platelet function under high shear stress

**Fig. 2:**
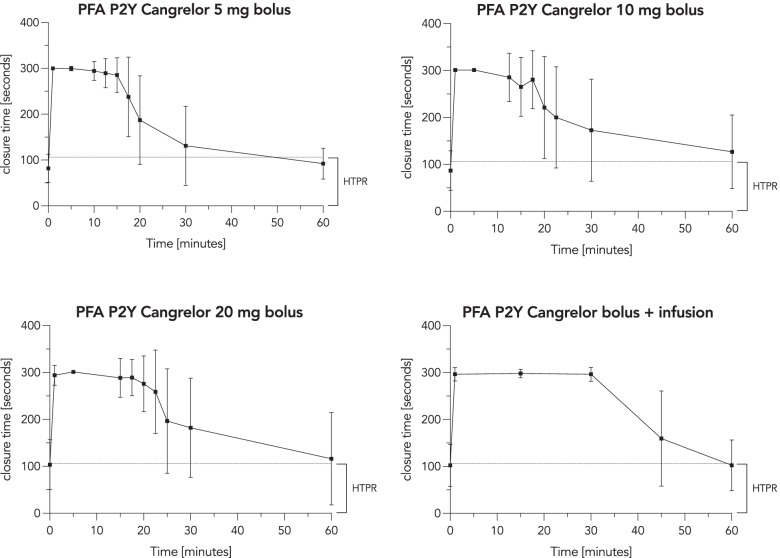
Platelet function under high shear rates. The four graphs show closure time (measured by the P2Y test of the PFA-100) over time following cangrelor bolus infusions of 5 mg (upper left panel, *n* = 12), 10 mg (upper right panel, *n* = 12), 20 mg (lower left panel, *n* = 11) and weight-adapted cangrelor bolus infusion followed by a continuous infusion via IV flow regulator (lower right panel, *n* = 11). The dashed line marks the recommended high on-treatment platelet reactivity threshold of 106 s. The PFA-100 does not measure closure times exceeding 301 s, therefore ranges are not shown for a better readability. Data are shown as mean ± standard deviation. (PFA-100 = platelet function analyzer)

Across all bolus infusions, closure time rapidly increased to > 301 s (measured with the PFA P2Y test). The antiplatelet effect under high shear stress was dose-dependent and lasted for 15 min, 17.5 min and 20 min in all subjects with bolus infusions of 5 mg, 10 mg and 20 mg, respectively. Cangrelor bolus infusion followed by continuous infusion via IV flow regulator resulted in pronounced and stable platelet inhibition. Platelet function under high shear stress returned to normal in 8 of 11 subjects 30 min after end of infusion (Fig. 2).

#### VASP-P assay

**Fig. 3:**
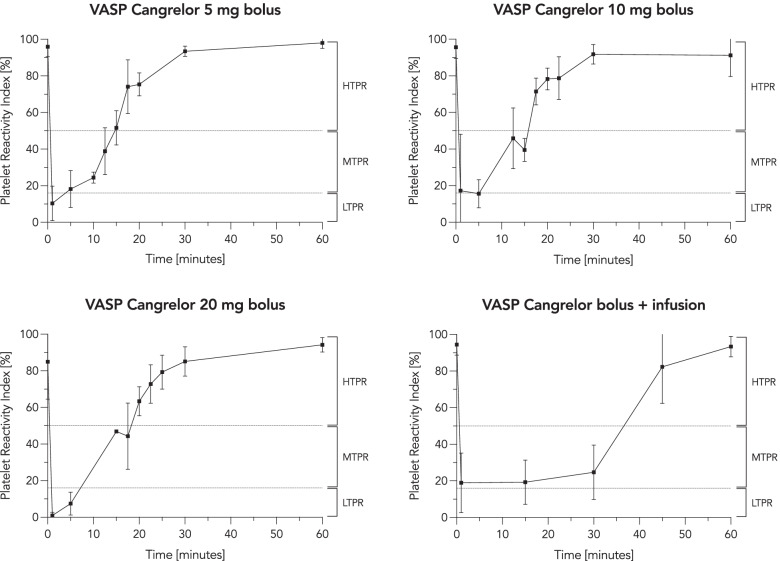
Vasodilator-stimulated phosphoprotein phosphorylation (VASP-P) assay. The four graphs show the platelet reactivity index [%] (measured by the VASP-P assay) over time following cangrelor bolus infusions of 5 mg (upper left panel, *n* = 12), 10 mg (upper right panel, *n* = 12), 20 mg (lower left panel, *n* = 11) and weight-adapted cangrelor bolus infusion followed by a continuous infusion via IV flow regulator (lower right panel, *n* = 11). The dashed line marks the recommended high on-treatment platelet reactivity threshold of PRI 50%. Data are shown as mean ± standard deviation

Pronounced platelet inhibition, as measured by the VASP-P assay, was achieved across all three bolus infusion groups. Sufficient platelet inhibition, defined as a platelet reactivity index of ≤50%, was achieved in all subjects for 10 min, 15 min and 15 min with bolus infusions of 5 mg, 10 mg and 20 mg, respectively. Bolus infusion of cangrelor followed by a continuous infusion via IV flow regulator resulted in immediate platelet inhibition at a mean PRI of 20%, which was maintained throughout the duration of infusion. Platelet reactivity returned to normal within 30 min after cessation of infusion (Fig. 3).

### Effect of ex vivo epinephrine on platelet function

Addition of epinephrine, in concentrations of 200 ng/mL and 500 ng/mL, to blood samples of subjects treated with cangrelor, resulted in a significant increase from a median ADP-induced platelet aggregation of 10 U to 30 U (*p* = 0.001) and 34 U (*p* = 0.002), respectively.

Likewise, epinephrine, when added in concentrations of 200 ng/mL and 500 ng/mL to blood samples of subjects under treatment with cangrelor, significantly increased the platelet reactivity index from a median of 24.4 to 61.8% (*p* = 0.002) and 61.9% (*p* = 0.0039), respectively.

**Fig. 4:**
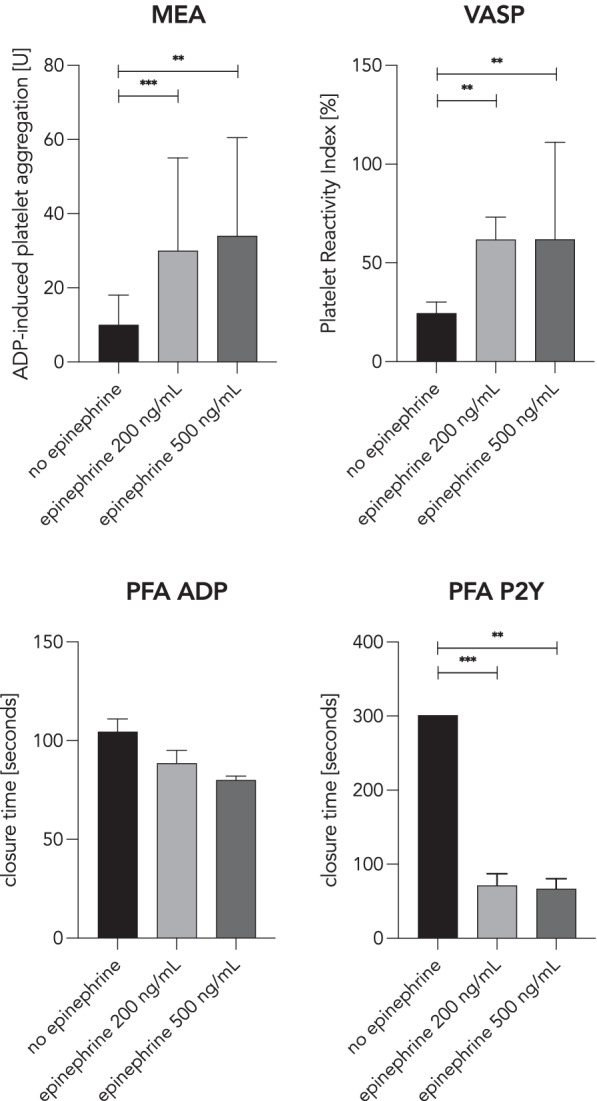
Antiplatelet reversal with epinephrine. An ex vivo experiment was performed to test the ability of epinephrine to reverse the antiplatelet effects of cangrelor. Each of the four graphs displays a different platelet function test (upper left: MEA (*n* = 11), upper right: VASP-P (*n* = 10), lower left: PFA ADP (*n* = 2), lower right PFA P2Y (*n* = 11)). The three columns in each graph represent the spiked concentration of epinephrine: no epinephrine added (left), epinephrine in a concentration of 200 ng/mL (middle) and epinephrine in a concentration of 500 ng/mL (right). Of note, in the experiment using the PFA ADP test, samples from just two subjects were tested and therefore no statistical significance was calculated. Two asterisks (**) describe a p value of ≤0.01, whereas three asterisks (***) describe a *p* value of ≤0.001. Data are shown as median and interquartile range

Epinephrine, in concentrations of 200 ng/mL and 500 ng/mL significantly shortened closure times from a median closure time of 301 s to 71 s (*p* = 0.001) and 66.5 s (*p* = 0.002), respectively, when measured with the P2Y test of the PFA-100. Results of the effect of ex vivo epinephrine on platelet function are shown in Fig. 4.

### Safety

Bolus infusions of up to 20 mg were safe and caused no adverse events, especially with regard to bleeding events. Vital signs did not change significantly in the active trial phase.

One trial subject (008) showed a prolonged antiplatelet effect to the second bolus infusion of cangrelor (10 mg). ADP-induced platelet aggregation (MEA) and closure time (tested with the PFA-100 P2Y test) returned to baseline only after around 230 min. Platelet inhibition measured by the VASP-P assay returned to baseline levels around 30 min (Supplementary Fig. S1). For safety reasons, we did not undertake the third bolus infusion and the weight-adapted bolus infusion followed by the continuous infusion.

## Discussion

This pilot trial in healthy volunteers investigated the antiplatelet effect of cangrelor bolus infusions and bolus infusion followed by a continuous infusion via IV flow regulator. Continuous infusion of cangrelor is usually performed using an electronic syringe pump, but such devices may not be universally available in ambulance units. Therefore, we tested the continuous infusion of cangrelor using an IV flow regulator, which allows for practicability and simplicity, two important factors in prehospital care. Cangrelor bolus infusions of 5 mg, 10 mg and 20 mg resulted in sufficient platelet inhibition for a duration of approximately 20 min when tested with MEA. Platelet function as assessed by the PFA-100 and the VASP-P assay responded in a similar manner.

The VASP-P assay specifically measures inhibition of the P2Y_12_ receptor by quantifying a downstream phosphorylation product [23]. It shows good reproducibility [24] and is predictive of stent thrombosis and major adverse cardiac events following coronary artery stenting [25, 26]. However, it may not accurately reflect platelet aggregation, as platelet aggregation is a complex process in which several receptors and signaling cascades interact, also independent of P2Y_12_ receptor activation. In terms of clinical significance, ADP-induced platelet aggregation may be considered a more practical method to assess platelet function because it reflects the result of platelet activation – platelet aggregation [27].

Cangrelor, when administered as a weight-adapted bolus (30 μg/kg) followed by a continuous infusion via an IV flow regulator (4 μg/kg/min), was easily feasible and resulted in immediate platelet inhibition, which was maintained for the duration of the infusion.

Bolus infusions of cangrelor work fine but show a high inter-subject variability in the MEA analysis. High variability in platelet reactivity and inconclusive dose-response relationships may be caused by a sequence effect or diurnal variation. A single bolus infusion of cangrelor 20 mg would necessitate a second one after approximately 20 min in order to maintain adequate platelet inhibition (≤46 U in MEA) for a longer period of time. Differently, a weight-adapted bolus infusion of cangrelor followed by continuous infusion using an IV flow regulator results in immediate and persistent platelet inhibition which can be reversed in less than 30 min after the infusion is stopped. We therefore conclude that, for prehospital use, the administration via the IV flow regulator presents a more attractive and feasible option.

Moderate on-treatment platelet reactivity (19-46 U) has been postulated as the recommended therapeutic window for the pharmacodynamic treatment response to P2Y_12_ inhibition after PCI [6, 28]. In our study, however, weight-adapted bolus infusion of cangrelor (30 μg/kg) followed by a continuous infusion (4 μg/kg/min) via IV flow regulator resulted in immediate and consistent LTPR. While a lower level of platelet reactivity could be considered advantageous in a state of acute thromboembolism, there is currently no evidence to support this theory, as LTPR does not further decrease the risk of ischemic events but leads to a higher risk of bleeding instead. Whether this pronounced antiplatelet effect translates to real-world patients remains to be elucidated.

The prehospital use of cangrelor in STEMI patients would implicate a couple of benefits. First, the use of cangrelor eliminates the need for oral intake of medication in the acute setting of STEMI, which is more practicable and reliable, especially in the setting of cardiogenic shock [29]. Second, intravenous P2Y_12_ inhibition allows to bypass the gastrointestinal tract, which can be affected by opioid treatment resulting in less drug uptake and a reduced pharmacodynamic response [11, 30]. Additionally, intravenous P2Y_12_ inhibition is unaffected by vomiting, which may also occur in STEMI patients (both as a symptom and as a consequence of opioid treatment). Third, cangrelor induces potent platelet inhibition but its antiplatelet effect is eliminated fast due to its short half-life. This particular characteristic allows for a well controllable and strong antiplatelet effect which enables flexibility. Conceivably, the use of cangrelor may allow patients with a coronary artery anatomy that requires coronary-artery bypass grafting to undergo surgery more rapidly [31].

The concept of periprocedural antiplatelet treatment with cangrelor has been tested before. Although the primary outcome (composite of death, myocardial infarction or ischemia-driven revascularization at 48 h after PCI) failed in the CHAMPION PCI [32] and the CHAMPION PLATFORM [33] trial, cangrelor significantly reduced the incidence of stent thrombosis (0.6 to 0.2%, *p* = 0.02) and death from any cause (0.7 to 0.2%, *p* = 0.02) at 48 h [33, 34]. In the CHAMPION PHOENIX trial, peri-procedural cangrelor significantly reduced the rate of death, myocardial infarction, ischemia-driven revascularization and stent thrombosis without increasing the rate of major bleeding at 48 h in patients undergoing urgent or elective PCI [35]. Granted, in all CHAMPION trials, cangrelor was compared to clopidogrel, which has nowadays almost completely been replaced by more potent P2Y_12_ inhibitors and is not routinely used in ACS patients undergoing PCI. In terms of timing of P2Y_12_ inhibitor administration, cangrelor was given in a periprocedural time frame in all three CHAMPION trials. In contrast, the current study aimed to develop an advanced treatment concept for effective prehospital antiplatelet therapy using cangrelor.

### Effect of epinephrine on platelet reactivity

Up to this day, there are no approved antidotes for P2Y_12_ inhibitors. An antibody-based ticagrelor reversal agent has been successfully tested in both healthy volunteers and patients but is not approved nor widely available [36, 37]. Antiplatelet reversal strategies for P2Y_12_ inhibitors are duly needed. Epinephrine induces platelet aggregation via the α_2_-adrenergic receptor, sharing the same downstream effects as the P2Y_12_ receptor [38]. The concept of epinephrine use as an antiplatelet reversal agent has been tested before in an in vitro model in ticagrelor-treated patients [39]. In our trial, epinephrine, in concentrations of 200 ng/mL and 500 ng/mL, spiked into blood from cangrelor-treated healthy volunteers significantly increased ADP-induced platelet aggregation in a dose-dependent manner, which further extends its antiplatelet-reversing ability from ticagrelor to cangrelor. Our chosen epinephrine concentrations were based on pharmacokinetic calculations of epinephrine during cardiac arrest [40, 41]. Our findings have two implications: (i) effective platelet inhibition may be particularly important in successfully resuscitated cardiac arrest patients with myocardial infarction as underlying cause [29], (ii) when measuring pharmacodynamic effects of P2Y_12_ inhibitors in patients who received epinephrine the results may be influenced. Thus, because of its platelet activating potential, the use of epinephrine in cardiac arrest might be unfavorable in patients with underlying coronary thrombosis, a phenotype often present in cardiac arrest patients [42].

However, the effect of epinephrine on platelet reactivity already happens at much lower concentrations, e.g. under continuous infusion [43], which might be of use in unstable, bleeding patients for additional control of hemostasis. This also supports the clinical use of local epinephrine infiltration in case of localized bleeding.

### Limitations

Our trial has several limitations. First, because this was a pilot trial, the sample size is rather small (*n* = 12). Second, our trial was conducted in healthy volunteers without any risk of bleeding, instead of real-world patients. This limits the applicability of our safety results to STEMI patients, who may have a significantly higher bleeding risk. Third, this is an open-label trial; the trial design could have been improved by conducting a double-blinded, randomized-controlled trial. Fourth, healthy volunteers were not pretreated with aspirin and unfractionated heparin, which could have helped to imitate the real-world treatment of STEMI.

## Conclusion

Both tested treatment regimens of cangrelor, fixed-dose bolus infusion and weight-adapted bolus infusion followed by a continuous infusion via IV flow regulator, resulted in immediate and pronounced platelet inhibition. Treatment with cangrelor caused no bleeding-associated adverse events. Cangrelor given as weight-adapted bolus infusion followed by a continuous infusion via IV flow regulator may present a feasible treatment strategy for effective prehospital platelet inhibition.

## Supplementary Information

**Additional file 1.**

## Data Availability

Additional data can be made available upon request by the corresponding author.
