# Supplementary material for: Advanced pharmacodynamics of cangrelor in healthy volunteers: a dose-finding, open-label, pilot trial
Source: Thromb J. 2022 Apr 14;20:19. doi: 10.1186/s12959-022-00377-z (PMC9008922; doi:10.1186/s12959-022-00377-z)
Supplement: Supplementary file 1 — Additional file 1. [file 12959_2022_377_MOESM1_ESM.docx]

**Advanced Pharmacodynamics of Cangrelor in Healthy Volunteers: a dose-finding, open-label, pilot trial**

**Rationale for cangrelor dosing**

The chosen bolus infusions of cangrelor were based on pharmacokinetic and pharmacodynamic experiments of cangrelor by *Akers et al.* 2010.

According to this study, a bolus infusion of cangrelor (30µg/kg) results in a maximum concentration (c_max_) of 635ng/mL and platelet inhibition is maintained for approximately 10 minutes after end of infusion. At a concentration of around 75ng/mL, platelet inhibition begins to decrease.

Pharmacokinetic calculations were performed for a healthy individual weighing 85kg. 75ng/mL was assumed as the threshold for maximum inhibition of platelet aggregation. The half-life of cangrelor was assumed to be 3.5 minutes. We assumed a c_max_ of 600ng/mL after a bolus infusion of 30µg/kg. Thus, a bolus infusion of 2.5mg would achieve sufficient platelet inhibition for 10.5 minutes. Consequently, cangrelor bolus infusions of 5mg, 10mg and 20mg would result in sufficient platelet inhibition for about 14, 17.5 and 21 minutes, respectively. Assuming a short time period of drug distribution, this may add a minute to the calculated time of effect.

A time window of 20-30 minutes of sufficient platelet inhibition was chosen, as this was deemed to be enough time to transport the patient to the nearest (PCI) hospital (in an urban area).

**Supplementary figure S1.** Time interval of platelet function/inhibition measured by multiple electrode aggregometry (MEA), platelet function analyzer 100 (PFA-100) and vasodilator-stimulated phosphoprotein phosphorylation assay (VASP-P) of the cangrelor 10 mg bolus infusion of trial subject 008.
